# The Effect of Corrective Feedback on Performance in Basic Cognitive Tasks: An Analysis of RT Components

**DOI:** 10.5334/pb.240

**Published:** 2016-12-20

**Authors:** Carmen Moret-Tatay, Craig Leth-Steensen, Tatiana Quarti Irigaray, Irani I. L. Argimon, Daniel Gamermann, Diana Abad-Tortosa, Camila Oliveira, Begoña Sáiz-Mauleón, Andrea Vázquez-Martínez, Esperanza Navarro-Pardo, Pedro Fernández de Córdoba Castellá

**Affiliations:** 1 Department of Neuropsychology, Methodology and Social Psychology, Universidad Católica de Valencia “San Vicente Mártir” C/Guillem de Castro, 175. Valencia 46008, ES; 2 Department of Psychology, Carleton University, 1125 Colonel By Drive, Ottawa, ON, Canada, CA; 3 Universidade Católica do Rio Grande do Sul, Faculdade de Psicologia, Programa de Pós-Graduação em Psicologia, BR; 4 Instituto de Física, Universidade Federal do Rio Grande do Sul (UFRGS), Av. Bento Gonçalves 9500, CP 15051, 91501-970 Porto Alegre RS, BR; 5 Facultat de Psicologia, Universitat de València, Avinguda Blasco Ibáñez, 21, ES; 6 Universitat Politècnica de València, Departamento de expresión gráfica arquitectónica and IUMPA Camino de Vera s/n, 46022, Valencia, ES

**Keywords:** feedback, stroop, matching task, ex-Gaussian components

## Abstract

The current work examines the effect of trial-by-trial feedback about correct and error responding on performance in two basic cognitive tasks: a classic Stroop task (*n* = 40) and a color-word matching task (*n* = 30). Standard measures of both RT and accuracy were examined in addition to measures obtained from fitting the ex-Gaussian distributional model to the correct RTs. For both tasks, RTs were faster in blocks of trials with feedback than in blocks without feedback, but this difference was not significant. On the other hand, with respect to the distributional analyses, providing feedback served to significantly reduce the size of the tails of the RT distributions. Such results suggest that, for conditions in which accuracy is fairly high, the effect of corrective feedback might either be to reduce the tendency to double-check before responding or to decrease the amount of attentional lapsing.

## Introduction

An important aspect of experimentation within cognitive psychological paradigms is whether or not to provide participants with trial-by-trial corrective feedback regarding the accuracy of their performance on cognitive tasks. As noted by Krenn, Würth, and Hergovich (2013), the provision of feedback can make individuals aware of any discrepancies between actual states of performance and target states, thus allowing for an evaluation of previous performance in relation to a specific standard.

Not surprisingly, the effect of feedback on human behavior has attracted the interest of cognitive researchers. A large number of studies have examined its effect on different variables such as motivation (Deci, Koestner, & Ryan, 1999; Jussim, Sofaleta, Brown, Law, & Kohlhepp, 1992) and learning (Goodman & Wood, 2004; Wulf, Shea, and Lewthwaite, 2010). However, not as much research has been directed at determining the role that corrective accuracy feedback plays in shaping performance on tasks that involve fairly basic perceptual- or cognitive-based decision making. Hence, such work is the focus of the current study.

### Some potential effects of feedback

One view of the effect of accuracy feedback on performance in basic cognitive tasks can be found in Starns and Ratcliff (2010). In their Experiment 2, participants saw a 10 × 10 grid of characters with some of the cells blank and others randomly filled by asterisks. The task was to quickly but accurately decide whether the number of asterisks displayed was either more or less than 50. The actual number of asterisks displayed on any trial ranged uniformly from 31 to 70. In a feedback condition, a post-trial correct or error message was displayed for 600 ms, and separate groups took part in feedback and no feedback conditions (i.e., the presence of feedback was manipulated in a between-participants fashion). When fitting the Ratcliff diffusion model to the obtained response time (RT) results, Starns and Ratcliff (2010) found that the major effect of providing feedback (for the college students but, interestingly, not for the aged 60+ seniors in their sample) was to lower the response criteria (i.e., the decision boundaries). In such a model, response criteria represent the amount of sequentially sampled evidence that must be accumulated in order to make a response (where the strength of the sampled evidence signal itself is referred to as the drift rate). Starns and Ratcliff (2010) conjectured that the provision of feedback helped the younger participants to be more aware of the potential for a balance between speed and accuracy, thus motivating them to make adjustments which then led to more optimal responding (i.e., lowering their response criteria which allowed them to respond faster with what could be regarded as minimal losses in accuracy). Indeed, follow-up analyses by these researchers indicated that the younger group were invoking response criteria when given feedback that were much closer to what Starns and Ratcliff (2010) referred to as the “reward-rate optimal boundary” than were the younger group who were not given feedback. Note that an analogous empirical result can be found in work by Appelgren, Penny, and Bengtsson (2014) involving the n-back memory task. In that study, accuracy-feedback-induced reductions in both RT and accuracy were observed but only for conditions in which feedback was provided after each correct response.

A second related view of the role of accuracy feedback on performance in basic cognitive tasks is that it affects strategy choice. Namely, that it aids participant’s determination of the optimality of the various possible processing strategies that could be applied to the current task. For example, Touron and Hertzog (2014) had participants perform a task that, after some degree of practice, could be solved using either a more time-consuming but highly accurate visual search strategy or a fast but occasionally error-prone memory retrieval strategy. They observed an increase in the trial-by-trial, self-reported use of memory retrieval for a group provided with accuracy feedback compared to a group with no feedback (although mainly for their older participants given that the younger participants were already employing quite high levels of memory retrieval). With respect to simple choice tasks involving highly overlearned and unbiased stimulus-response associations, such as the color-naming and matching tasks examined in the present study, one tempting but quite inefficient processing strategy would be to periodically double-check the stimulus display before making a response. Given that accuracy is typically fairly high to begin with in tasks of this nature, the provision of accuracy feedback could signal that such double-checking is not enhancing performance that much and, hence, could perhaps be dropped (which would then serve to speed up the RTs for all of the trials in which such double-checking would have occurred but didn’t and also to potentially reduce accuracy somewhat, given less double-checking).

A third related view of the role of accuracy feedback on performance in basic cognitive tasks is that it affects the amount and focus of the effort devoted to a task (Krenn et al., 2013). In the context of the current research, in the first upcoming study, performance on the classic Stroop task is examined. In a Stroop task, more mistakes are generally made on incongruent Stroop trials. Hence, the provision of corrective feedback when performing such a task could make participants more aware of the fact that the irrelevant stimulus attribute is having an effect on responding and that more effort needs to then be devoted to minimizing the processing of it (either by focusing more intently on selecting the relevant attribute or by attempting to supress all processing of the irrelevant one). Such a view is also in line with the conflict-monitoring account of cognitive control (Botvinick, Braver, Barch, Carer, & Cohen, 2001) which posits that the presence of enhanced stimulus-response conflict in a task such as the Stroop task signals the need for greater cognitive control. Such control can be envisioned in terms of a top-down attentional biasing of the relevant stimulus attribute that would then serve to enhance the strength of the evidence signal (i.e., drift rate) resulting in faster and, potentially also, more accurate responding. This account is able to provide an explanation for the finding that Stroop interference is reduced in blocks of trials containing a greater proportion of incongruent trials. With respect to the role of corrective feedback in the Stroop task, such feedback could be assumed to provide an explicit signal for the need for greater cognitive control (Bugg & Smallwood, 2014). Moreover, with respect to other types of basic cognitive tasks that do not involve such conflict, note that it would not generally be that hard to envision conditions under which an enhanced marshalling of attentional resources would lead to stronger evidence signals.

A fourth related view of the role of accuracy feedback on performance in basic cognitive tasks is that it affects the deployment of attentional resources more generally. On one hand, the provision of feedback could serve to divert attention away from the performance of the task by capturing limited attentional resources (MacLeod & MacDonald, 2000). Under such conditions, though, it could then be assumed that both accuracy and RT performance would suffer (i.e., lower accuracy accompanied by slower RTs when feedback has been provided). On the other hand, also in line with the notion that feedback enhances effort, the provision of feedback might serve to prompt participants to cut down on the number of attentional lapses that can occur when repeatedly performing such simple tasks either by enhancing motivation or increasing arousal/activation levels (for a related view see Kole, Healy, & Bourne, 2008). Cutting down on attentional lapses would reduce the number of both very slow correct responses and attentional-based errors (although note that if such errors are rather infrequent to begin with, increases in accuracy due to reductions in the number of attentional lapses might not actually turn out to be that substantial). One bit of evidence for this view can actually also be found in the RT modeling work done by Starns and Ratcliff (2010) on the results from their Experiment 2 (detailed above). Namely, the version of the diffusion model that they used to fit the RTs also contained, what was referred to as, a “proportion of RT contaminants” parameter (i.e., *p_o_*) which was specifically assumed by them to represent lapses in attention. On the proportion of trials indicated by the value of this parameter, an additional response delay, taken from a uniform probability distribution, was assumed to be added to the corresponding diffusion model latency. Interestingly, although never actually addressed by Starns and Ratcliff (2010), the estimated value of this parameter was lower when feedback was provided than when it was not (i.e., 0.2% vs. 1.8% of trials, respectively).

### Ex-Gaussian distributional model

At this point, it is important to focus on the dependent variables that might serve to best shed light on feedback effects. Of course, measures of performance accuracy and RT will be key in this respect. However, one important way to extend typical analyses of proportion correct (PC) and mean RT is to examine the distributions of RTs obtained as a whole by fitting distributional models such as the ex-Gaussian (Balota & Spieler, 1999; Heathcote et al., 1991). The ex-Gaussian function represents the convolution of two probability distribution functions: A Gaussian (i.e., normal) and an exponential. This function generally serves to capture, quite adequately, the positively skewed shapes of most RT distributions and it can be quantified using three parameters: μ (the mean of the normal component), σ (the standard deviation of the normal component), and τ (both the mean and standard deviation of the exponential component). In a more intuitive vein, the first two parameters (i.e., μ and σ) serve to summarize the location of the leading edge of a distribution of RTs, whereas the third parameter (i.e., τ) provides summary information related to the size of the tail of such distributions.

Importantly, like RT itself, the μ and τ parameters have been shown to be remarkably sensitive to the effects of various types of cognitive-based experimental manipulations (for a review see Matze & Wagenmakers, 2009; and see also Kristjánsson, & Jóhannesson, 2014; Leth-Steensen, 2009; Moreno-Cid et al., 2015; Moret-Tatay et al., 2014; Navarro-Pardo, Navarro-Prados, Gamermann, & Moret-Tatay, 2013). However, as extensively discussed by Matzke and Wagenmakers (2009), the substantive interpretation of the ex-Gaussian parameters is still being debated and varies considerably among researchers and theories. The key issue in this regard is the extent to which changes in individual ex-Gaussian parameters can exclusively be attributed to the effects of experimental manipulations on specific cognitive processes such as decisional versus sensory/motor processes or automatic versus controlled processes (Matze & Wagenmakers, 2009).

Nonetheless, the accounts mentioned above regarding the potential effects of providing accuracy feedback do provide some testable predictions regarding changes in the ex-Gaussian parameters μ and τ that would be expected due to the provision of feedback. First, if providing feedback leads to downwards adjustments of decision criteria to achieve a more optimal balance between the speed and accuracy of responding such adjustments, as clearly demonstrated by Matzke and Wagenmakers (2009; see also Smith & Mewhort, 1998), would be expected to lead to decreases in both μ and τ for conditions in which feedback has been provided in comparison to conditions in which it has not. Second, if providing feedback leads to attentional-based strengthening of evidence signals due to enhancements in cognitive control, then such strengthening would also be expected to lead to decreases in both μ and τ for conditions in which feedback has been provided in comparison to conditions in which it has not (Matzke & Wagenmakers, 2009; Smith & Mewhort, 1998; Spieler, Balota, & Faust, 2000; although one caveat is that, as demonstrated by Smith & Mewhort, the effect of increased signal strength, i.e., drift rate, on μ is not that dramatic for cases in which decision criteria are set fairly low).

Third, if the provision of feedback serves to induce a reduction in the number of trials in which a double-checking strategy might have been employed, such reductions would likely be expected to be reflected mainly in smaller values of the ex-Gaussian τ parameter. Such an expectation is in line with work by Penner-Wilger, Leth-Steensen, and LeFevre (2002) who found that the RT distributions obtained from Canadian graduate students when solving single-digit multiplication problems differed from those of Chinese graduate students mostly with respect to the size of τ (much larger for the Canadians). They concluded that this result was consistent with the fact that Canadian students are much more likely to report periodically using less efficient, non-retrieval solution strategies (as opposed to direct memory retrieval) than are Chinese students.

Finally, if the provision of feedback serves to induce a reduction in the number of attentional lapses, such reductions would also be expected to be reflected mainly, if not exclusively, in smaller values of the ex-Gaussian τ parameter. For example, Leth-Steensen, King Elbaz, and Douglas (2000) found that differences in the choice RTs obtained from ADHD and control children were reflected mainly in the tails of the RT distributions (i.e., in τ). They then conjectured that this effect was the result of an enhanced tendency for attentional lapsing in ADHD children (which would then lead to a larger proportion of very slow responses present in the tails of their RT distributions). In this same vein, for a sample of ADHD and typically developing adolescents, Gu, Gau, Tzang, and Hu (2016) found that, with respect to performance on the Connors continuous performance task, the ex-Gaussian parameter τ was highly positively correlated with the number of omission errors (i.e., misses) made by their participants. Such errors were higher for the ADHDs and high rates of such errors were regarded by Gu et al. (2016) as representing markers of attentional impairment. Finally, Spieler, Balota, and Faust (1996) observed enhanced Stroop effects for older adults that were due mostly to increases in tau. They then specifically attributed this result to the possibility that older adults might be more susceptible to experiencing attentional lapses on a greater proportion of trials than young adults.

### The current study

In the current work, RT and accuracy performance in both a Stroop color-word task (in Study I) and a subsequent color-word matching task (in Study II) is examined under conditions in which accuracy feedback is provided after each trial and under conditions in which no accuracy feedback is provided. The first task is one of the most broadly applied paradigms in cognitive psychology (Heathcote, Popiel, & Mewhort, 1991), and it has been related to selective attention, processing speed, cognitive flexibility, and executive functions (Howieson, Lezak, & Loring, 2004; Servant, Montagnini, & Burle, 2014). In its standard set up, the Stroop paradigm requires participants to identify the font color of presented color words while trying to ignore congruent and incongruent color names. For the second task, the element of potential conflict between irrelevant aspects of the stimuli and the responses was eliminated by having participants perform a color-word matching task. Namely, they were simply asked to indicate if word names were congruent or not with the color of the font that the words were presented in. One important key aspect of the current work is that in addition to standard analyses involving mean RT and PC, RT distributional analyses are also performed by fitting the ex-Gaussian distributional model to the distributions of RTs obtained from individual participants in each condition of Studies I and II.

## Method

### Participants

A sample of 40 University students volunteered to take part in Study I (29 women and 11 men with an average age of 20.02 years and SD = 1.22). A sample of 30 students, from the same University, volunteered to take part in Study II (26 women and 4 men with an average age of 20.27 years and SD = 1.26). All the participants had normal vision or corrected to normal, were native Spanish speakers, and did not report any cognitive or neurological disorders.

### Materials

The presentation of the stimuli and recording of RT and PC were controlled by computers through the Windows software DMDX (Forster & Forster, 2003). On each trial, a fixation point (+) was presented for 500 ms in the center of the screen. Then the target stimulus was presented until the participants responded, with a maximum of 2500 ms given to respond. Word stimuli in Study I were *rojo* (which means red), *azul* (which means blue), and *xxxx*. Word stimuli in Study II were *rojo* and *azul*. The stimuli were presented in lowercase 14-pt Courier.

### Design

In Study I, a 2 (presence and absence of feedback) × 3 (congruent, incongruent, and control Stroop conditions) factorial design was used. Participants were required to identify the color red by pressing one key and the color blue by pressing another. The stimuli was presented in either red or blue uppercase letters, and there were three presentation conditions: i) Congruent - red word displayed in red or blue word displayed in blue, ii) incongruent - red word displayed in blue or blue word displayed in red, and iii) neutral - xxxx in red or blue. Participants performed 18 practice trials and 240 experimental trials.

In Study II, a 2 (presence and absence of feedback) × 2 (congruent and incongruent matching conditions) factorial design was used. Participants were required to identify if the font color was congruent with the written word by pressing one key, or not, by pressing another key. This is why condition iii) from Study I was not possible for Study II. The stimuli were presented in either red or blue lowercase letters, and there were two presentation conditions: i) Matching - red word displayed in red or blue word displayed in blue, and ii) nonmatching - red word displayed in blue or blue word displayed in red. Participants performed 18 practice trials and 160 experimental trials.

For both studies, the stimuli were presented in a randomized fashion within blocks. Furthermore, participants from each study were divided into two groups. The first group started with a block which provided immediately informative feedback and a following block which did not. The second group performed the task with the order of these blocks reversed. Informative feedback was provided immediately after each participant’s response that indicated whether it was *Correcto* (correct) or *Error* (error). RTs to perform the task on each trial were recorded in milliseconds. Each session lasted about 20 minutes.

### Analysis

The dependent measures to be analysed in each study were the (arcsine-transformed) PCs, mean correct RTs, and the values of each of the three ex-Gaussian parameters μ, σ, and τ. Values for each of these measures were obtained for each participant individually for each condition in the corresponding designs (i. e., the ex-Gaussian model was fit separately for each participant to the sets of correct RTs obtained in each of the six conditions of Study I and the four conditions of Study II). Any correct RTs shorter than 150 ms or more than 4 SDs above the mean in each condition (Leth-Steensen et al., 2000) were removed before running the RT analyses (i.e., 0.7% of the Study I correct RTs and 0.9% of the Study II correct RTs).

The software used to perform the ex-Gaussian fits made use of quantile maximum probability estimation (QMPE; Heathcote, Brown, & Cousineau, 2004). QMPE fits the ex-Gaussian distribution to a set of quantile values that have been estimated from a set of RT data. Directly analogous to percentile values, quantiles represent the RT value for which a certain proportion of observed RTs fall below it. Essentially, what QMPE is doing is invoking a search for the values of μ, σ, and τ that result in ex-Gaussian-based quantile values that most closely match the actual quantile values in a set of RT data. This search is undertaken using maximum likelihood techniques. More specifically, the best fitting ex-Gaussian curve is associated with the three ex-Gaussian parameter values for which the likelihood of the observed set of actual quantile values is a maximum. For the present fits, the number of quantiles used was set at 10.

## Results

### Study I

Average RTs for correct responses and PCs are presented in Table 1. A 2 (feedback condition) × 3 (Stroop condition) fully repeated measures ANOVA was performed on both the RTs and PCs.

**Table 1:** Mean RTs (in ms) and PCs for the Stroop and Feedback Conditions in Study I.

	Congruent	Incongruent	Neutral

RT			
Feedback	443	468	447
No Feedback	462	490	459
PC			
Feedback	0.983	0.954	0.976
No Feedback	0.974	0.959	0.976

As expected, RT showed Stroop effects: *F*(2, 78) = 21.00, *p* < .001, *MSE* = 888, η*_p_*^2^ = .350. On the other hand, although RTs were faster for the feedback condition than for the no-feedback condition for all three Stroop conditions, this difference was not significant at the conventional .05 level: *F*(1, 39) = 3.08, *p* < .10, *MSE* = 5940, η^2^ = .073. With respect to the PCs, significant Stroop effects were also present: *F*(2, 78) = 11.70, *p* < .001, *MSE* = 0.018, η*_p_*^2^ = .231. However, neither the main effect of the feedback conditions nor its interaction with the Stroop conditions were statistically significant (*F*s < 1).

**Figure 1:**
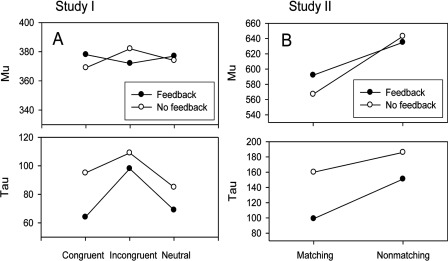
Values of the ex-Gaussian parameters μ (i.e., mu) and τ (i.e., tau) at each feedback by Stroop condition in Study I **(1A)** and at each feedback by matching condition in Study II **(1B)**.

With respect to the three ex-Gaussian parameters, analogous ANOVAs were performed on each of them as well (plots of the effects present in μ and τ are given in (Figure 1A). For μ, no main effects or interactions involving it were statistically significant (all *p*s > .10). For σ, as well, no main effects or interactions involving it were statistically significant (all *p*s > .10). For τ, however, both the main effect of the Stroop conditions, *F*(2, 78) = 12.29, *p* < .001, *MSE* = 1412, η*_p_*^2^ = .240, and the main effect of the feedback conditions, *F*(1, 39) = 11.66, *p* < .01, *MSE* = 1930, η^2^ = .230, were significant. Note, as well, that when the order in which the feedback was provided (i.e., first or second) was entered as a factor in the design, no significant main effects or interactions involving it were present for analyses involving either μ or τ (all *p*s > .05).

### Study II

Average RTs for correct responses and PCs are presented in Table 2. A 2 (feedback condition) × 2 (matching condition) fully repeated measures ANOVA was performed on both the RTs and PCs.

**Table 2:** Mean RTs (in ms) and PCs for the Matching and Feedback Conditions in Study II.

	Match	No match

RT		
Feedback	693	787
No Feedback	728	827
PC		
Feedback	0.940	0.953
No Feedback	0.961	0.953

As expected, RT showed matching effects: *F*(1, 29) = 167.07, *p* < .001, *MSE* = 1670, η*_p_*^2^ = .852. On the other hand, although RTs were faster for the feedback condition than for the no-feedback condition for each matching conditions, this difference was again not significant at the conventional .05 level: *F*(1, 29) = 3.96, *p* < .10, *MSE* = 10554, η^2^ = .120. With respect to the PCs, no main effects or interactions were statistically significant (all *p*s > .05).

With respect to the three ex-Gaussian parameters, analogous ANOVAs were performed on each of them as well (plots of the effects present in μ and τ are given in (Figure 1B). For μ, only the main effect of matching conditions was significant, *F*(1, 29) = 37.47, *p* < .001, *MSE* = 2800, η*_p_*^2^ = .564. For σ, no main effects or interactions involving it were statistically significant (all *p*s > .05). For τ, both the main effect of the matching conditions, *F*(1, 29) = 10.04, *p* < .01, *MSE* = 4398, η*_p_*^2^ = .257, and the main effect of the feedback conditions, *F*(1, 29) = 8.90, *p* < .01, *MSE* = 7767, η^2^ = .235, were significant. Note, as well, that when the order in which the feedback was provided (i.e., first or second) was entered as a factor in the design, no significant main effects or interactions involving it were present for analyses involving either μ or τ (all *p*s > .10).

## Discussion

The present results are clear in demonstrating that, for the two basic cognitive tasks examined here, providing accuracy feedback served to significantly reduce the size of the tails of the RT distributions only. That is, in both Studies I and II, although decreases in τ were observed when feedback was given, no such decreases in μ occurred. Highlighting the importance of obtaining a more detailed description of RT through an examination of the separate ex-Gaussian RT components, in both studies, the effect of feedback on overall RT was not significant at the .05 level. Hence, if only RTs had been examined here, any conclusions concerning the effect of providing feedback on the speed of responding would have to have been regarded as being somewhat tentative. Moreover, in neither study did the provision of such feedback serve to significantly affect the accuracy of responding (for further discussion of the potential effects of corrective feedback on overall accuracy see Appelgren et al., 2014, and for a demonstration of the fact that corrective feedback does not seem to affect post-error slowing see Houtman, Castellar, Notebaert, & Nu, 2012).

Importantly, the presence of feedback effects in τ but not in μ is somewhat diagnostic regarding the different views of the potential effects of such feedback discussed earlier. Namely, such a result is highly suggestive of the fact that providing feedback served to reduce either the tendency to double-check before responding or the amount of attentional lapsing. Given such possibilities, one aspect of future research might be to examine the role that various kinds individual difference variables such as anxiety (Bishop, Duncan, Brett, & Lawrrence, 2004; Henderson, Synder, Gupta, & Banich, 2012), might have in determining this feedback effect. Although the possibility that the present changes in τ with feedback were due to stronger evidence signals cannot be completely ruled out note that, as discussed earlier, small or negligible effects in μ under such conditions would only be expected if decision thresholds were set quite low (as they would be if participants had been instructed to emphasize speed), and there is no indication in either the RTs or the PCs from either Study I or II that this would have been the case here.

With respect to the first view regarding the potential effect of feedback discussed earlier, it seems that participants in the present studies were likely already performing quite optimally in terms of balancing out the speed and accuracy of their responding. Note that in Starns and Ratcliff’s (2010) Experiment 2, the discrimination task was actually rather difficult on some trials (e.g., deciding whether a display with 49 asterisks had either more or less than 50 asterisks) leading to an overall observed accuracy of around 85% correct that was much lower than in the present studies (as was also the case for the n-back memory task of Appelgren et al., 2014). Hence, such circumstances may have induced an overly cautious response set that was then subject to adjustments when feedback was provided (at least for their younger participants).

Finally, what might the results of this study have to say to cognitive researchers in general with respect to the issue of whether corrective accuracy feedback should or should not be included as part of their experimental procedures? Well, for studies that are concerned mainly with examining RT for which accuracy is typically quite high (i.e., > 90%), the current results suggest that providing such feedback would indeed tend to reduce the size of the tails of the RT distributions (hence, serving to lower both the RT means and standard deviations of individual participants). However, as is also evident in the current results, the effect of providing feedback did not interact with either of the other experimental manipulations (i.e., Stroop congruency or color-word match). If it is indeed the case that such feedback effects do not serve to moderate any other effects then, perhaps, the slight increases in the tails of the RT distributions that would occur when not providing such feedback might be something that could easily be tolerated. On the other hand, for those researchers who might then be interested in going on to fit parameterized process-based models, such as the diffusion model, to their RT data, reducing the possibility of either double-checking or attentional lapses (both of which could actually be regarded as RT contaminants) by providing corrective accuracy feedback might indeed be helpful with respect to achieving the best possible parameter estimates from those model fits.

## References

[1] Appelgren A., Penny W., Bengtsson S. L. (2014). Impact of feedback on three phases of performance monitoring. Experimental Psychology.

[2] Balota D. A., Spieler D. H. (1999). Word frequency, repetition, and lexicality effects in word recognition tasks: Beyond measures of central tendency. Journal of Experimental Psychology: General.

[3] Bishop S., Duncan J., Brett M., Lawrence A. D. (2004). Prefrontal cortical function and anxiety: controlling attention to threat-related stimuli. Nature Neuroscience.

[4] Botvinick M. M., Braver T. S., Barch D. M., Carer C. S., Cohen J. D. (2001). Conflict monitoring and cognitive control. Psychological Review.

[5] Deci E. L., Koestner R., Ryan R. M. (1999). A meta-analytic review of experiments examining the effects of extrinsic rewards on intrinsic motivation. Psychological Bulletin.

[6] Goodman J. S., Wood R. E. (2004). Feedback specificity, learning opportunities, and learning. Journal of Applied Psychology.

[7] Heathcote A., Brown S., Cousineau D. (2004). QMPE: Estimating Lognormal, Wald, & Weibull RT distributions with a parameter-dependent lower bound. Behavior Research Methods, Instruments, & Computers.

[8] Heathcote A., Popiel S. J., Mewhort D. J. K. (1991). Analysis of response time distributions: An example using the Stroop task. Psychological Bulletin.

[9] Henderson R. K., Snyder H. R., Gupta T., Banich M. T. (2012). When does stress help or harm? The effects of stress controllability and subjective stress response on Stroop performance. Frontiers in Psychology.

[10] Houtman F., Castellar E. N., Notebaert W., Nu E. (2012). Orienting to errors with and without immediate feedback. Journal of Cognitive Psychology.

[11] Howieson D. B., Lezak M. D., Loring D. W. (2004). Orientation and attention.

[12] Jussim L., Soffin S., Brown R., Ley J., Kohlhepp K. (1992). Understanding reactions to feedback by integrating ideas from symbolic interactionism and cognitive evaluation theory. Journal of Personality and Social Psychology.

[13] Kole J. A., Healy A. F., Bourne L. E. (2008). Cognitive complications moderate the speed-accuracy tradeoff in data entry: A cognitive antidote to inhibition. Applied Cognitive Psychology.

[14] Kristjánsson Á., Jóhannesson Ó. I. (2014). How priming in visual search affects response time distributions: Analyses with ex-Gaussian fits. Attention, Perception, & Psychophysics.

[15] Leth-Steensen C. (2009). Lengthening fixed preparatory durations within a digit magnitude classification task serves mainly to shift distributions of response times upwards. Acta Psychologica.

[16] Leth-Steensen C., King Elbaz Z., Douglas V. I. (2000). Mean response times, variability, and skew in the responding of ADHD children: a response time distributional approach. Acta Psychologica.

[17] MacLeod C., MacDonald P. (2000). Interdimensional interference in the Stroop effect: Uncovering the cognitive and neural anatomy. Trends in Cognitive Sciences.

[18] Matzke D., Wagenmakers E.-J. (2009). Psychological interpretation of ex-Gaussian and shifted Wald parameters: A diffusion model analysis. Psychonomic Bulletin & Review.

[19] Moreno-Cid A., Moret-Tatay C., Irigaray T. Q., Argimon I. I., Murphy M., Szczerbinski M., Fernández P. (2015). The Role of Age and Emotional Valence in Word Recognition: An Ex-Gaussian Analysis. Studia Psychologica.

[20] Moret-Tatay C., Argimon I., Quarty T., Moreno A., Szczerbinski M., Murphy M., Vázquez-Molina J., Vázquez-Martínez A., Saíz-Mauleon B., Navarro-Pardo E., Fernández de Córdoba Castellá P. (2014). The effects of age and emotional valence on recognition memory: ex-Gaussian components analysis. Scandinavian Journal of Psychology.

[21] Navarro-Pardo E., Navarro-Prados A. B., Gamermann D., Moret-Tatay C. (2013). Differences between young and old university students on lexical decision task: evidence through an ex-Gaussian approach. Journal of General Psychology.

[22] Penner-Wilger M., Leth-Steensen C., LeFevre J. (2002). Decomposing the problem-size effect: A comparison of response time distributions across cultures. Memory & Cognition.

[23] Servant M., Montagnini A., Burle B. (2014). Conflict tasks and the diffusion framework: Insight in model constraints based on psychological laws. Cognitive Psychology.

[24] Smith D. G., Mewhort D. J. K. (1998). The distributions of latencies constrains theories of decision time: A test of the random-walk model using numeric comparison. Australian Journal of Psychology.

[25] Spieler D., Balota D., Faust M. (1996). Stroop performance in healthy younger and elder adults and in individuals with dementia of the Alzheimer’s type. Journal of Experimental Psychology: Human Perception and Performance.

[26] Spieler D. H., Balota D. A., Faust M. E. (2000). Levels of selective attention revealed through analyses of response time distributions. Journal of Experimental Psychology: Human Perception and Performance.

[27] Starns J. J., Ratcliff R. (2010). The effects of aging on the speed-accuracy compromise: Boundary Optimality in the Diffusion Model. Psychology and Aging.

[28] Touron D. R., Hertzog C. (2014). Accuracy and speed feedback: Global and local effects on strategy usage. Experimental Aging Research.

[29] Wulf G., Shea C., Lewthwaite R. (2010). Motor skill learning and performance: a review of influential factors. Medical Education.

